# Reaction of rat connective tissue to mineral trioxide aggregate and diaket

**DOI:** 10.1186/1472-6831-11-17

**Published:** 2011-05-13

**Authors:** Wael M Al-Omari, Maisoun S Abu-Zaghlan, Huda M Hammad

**Affiliations:** 1 Associate Professor, Department of Conservative Dentistry, Faculty of Dentistry, Jordan University of Science and Technology, Irbid, Jordan; 2 Endodontist, Private sector, Amman, Jordan; 3 Associate Professor, Department of Oral Medicine and Surgery, Faculty of Dentistry, Jordan University of Science and Technology, Irbid, Jordan

## Abstract

**Background:**

The aim of this study was to compare the reaction of rat connective tissue to two root-end filling materials: white Mineral Trioxide Aggregate (WMTA) and Diaket.

**Methods:**

Each of the materials was placed in dentine tubes and implanted subcutaneously in the dorsal connective tissue of 21 Wistar albino rats. Tissue biopsies were collected 7, 30, and 60 days after the implantation procedure. The specimens were processed and stained with hematoxylin and eosin and examined microscopically. After determining inflammatory cell numbers in sections from each specimen, inflammatory reaction scores were defined as follows: 0; no or few inflammatory cells (no reaction), 1; less than 25 cells (mild reaction), 2; 25 to 125 cells, (moderate reaction), and 3; 125 or more cells (severe reaction). Statistical analysis was performed using the Kruskal-Wallis and Mann-Whitney tests.

**Results:**

There were statistically significant differences in the median inflammatory cell numbers throughout the three test periods, with the most severe degree of inflammation observed at the one-week period. Few cases of necrosis were observed with WMTA. Diaket exhibited the most severe degree of inflammation and necrosis. After 30 days, both materials provoked moderate inflammatory reaction. The eight-week period showed the least severe degree of inflammation in all groups.

**Conclusions:**

It was concluded that WMTA exhibits a more favourable tissue response compared with Diaket which induced more severe inflammatory reaction than WMTA and the control.

## Background

A periapical surgical procedure usually involves root-end resection, preparation and sealing, using a root-end filling material. Success of the surgery may depend on the sealing ability of the root-end filling material to prevent ingress of bacteria or their by-products, and also on its ability to provide a favourable environment for the regeneration and healing of periradicular tissues [1,2].

Mineral trioxide aggregate (MTA) was developed as a root-end filling material at Loma Linda University, California, USA [1]. The principal compounds in this material are tricalcium silicate, tricalcium aluminate, tricalcium oxide and silicate oxide [2]. *In vivo *and *in vitro *studies supported the potential use of this material as a root-end filling material [3-5]. Previous investigations also demonstrated the ability of MTA to encourage hard tissue deposition [3,6,7], and its good sealing ability compared to other materials [3,8-10]. Moreover, MTA was found to be well tolerated and biocompatible [11-17], and revealed a more favourable periapical tissue response compared with amalgam, IRM, and Super EBA [4,5,18].

Diaket is a calcium chelate reinforced with polyvinyl resin [19]. It was initially formulated as a root canal sealer when mixed in a powder to liquid ratio of 1:1. Tetsch, in 1986, first documented the use of Diaket as a root-end filling material when mixed to a thicker consistency [20]. Diaket demonstrated acceptable properties [21-23]. It demonstrated a superior seal compared to amalgam [23], a favourable healing potential in dogs [22], and a better healing response compared to gutta-percha [24].

Both Diaket and MTA were found to promote periradicular tissue regeneration. However, the handling properties of Diaket were considered to be superior to those of MTA [21].

The aim of the present study was to compare the reaction of rat connective tissue to the two root-end filling materials: white Mineral Trioxide Aggregate (WMTA) and Diaket.

## Methods

Twenty one male, 5-6 month-old Wistar Albino rats weighing 350 ± 50 g were used. Animal care was carried out according to the Institutional Animal Care and Use Committee and the Canadian Council on Animal Care [25,26]. The protocol was approved by the Jordan University of Science and Technology Deanship of Research, and the University Animal Care and Use Committee. The following groups were investigated:

Group 1: Control group (empty dentine tubes).

Group 2: White Mineral Trioxide Aggregate (Dentsply, Tulsa Dental, Tulsa, Ok, USA).

Group 3: Diaket, (3 M ESPE AG, Seefeld, Bayern, Germany).

Dentine tubes were prepared from human tooth roots of single rooted teeth at a length of 7 mm. The canals were enlarged using protaper files (Dentsply Maillefer, Ballaigues, Switzerland) and Gates Glidden burs (Dentsply Maillefer, Ballaigues, Switzerland) to a thickness of the outer wall of 0.5-1 mm. The apical foramina were enlarged into an approximate diameter of 2 mm. The dentine tubes were copiously irrigated with 3% sodium hypochlorite and 18% EDTA (ethylene diamine-tetra-acetic acid), (Ultra Dent, USA), washed with distilled water, then autoclaved. For each animal, three tubes were used, two as carriers of the two tested materials and an empty tube as a control.

WMTA was mixed according to the manufacturer's recommendations. Diaket was mixed to a consistency (powder to liquid ratio of 2:1) thicker than the manufacturer's recommendations, [21]. The materials were applied into the tubes.

The animals were anaesthetized using a regimen consisting of atropine (0.02-0.05 mg/kg), ketamine hydrochloride (40-87 mg/kg), and xylazine hydrochloride (5-13 mg/kg). The dorsal skins of the animals were shaved and disinfected with 5% iodine solution. Two centimeter long incisions were made in the back of each animal, at least 2 cm away from each other, using a no. 15 blade (Aesculap AG and Co. KG, Tutlingen, Germany) in a head to tail orientation. The skin was reflected and subcutaneous spaces were created by blunt dissection. The dentine tubes were immediately implanted into the spaces. The skin was closed with 3/0 silk suture.

Evaluations were made at 7, 30, and 60 days after surgical implantation. In each evaluation period, 7 animals were sacrificed using an anaesthetic overdose. The dorsal skin was shaved and the tubes were excised with the surrounding connective tissue. The excised specimens were kept in 10% formalin solution for 2 weeks. After tissue processing and embedding, serial sections of 6 μm thickness were prepared and stained with hematoxylin and eosin. Sections were examined and evaluated using a light microscope (Olympus, U-MDOB BX 40, Olympus optical Co. LTD, Japan) at ×40, ×100, and ×400 magnifications. For each material, the sum and average of inflammatory cells (polymorphonuclear leukocytes (PMNs), plasma cells, lymphocytes, macrophages and giant cells) and fibroblasts were determined in ten separate areas at ×400 magnification. The observer was blinded to the tissue source.

Reactions in the tissue were scored as: 0, none or few inflammatory cells (no reaction); 1, less than 25 cells (mild reaction); 2, between 25 and 125 cells (moderate reaction); and 3, 125 or more cells (severe reaction), [13,27,28]. Necrosis and/or suppuration were categorized as "yes" or "no".

Results were statistically analyzed using the Kruskal-Wallis and Mann-Whitney tests for comparison of severity of inflammation at intervals and for materials.

## Results

### Control group

**Figure 1:**
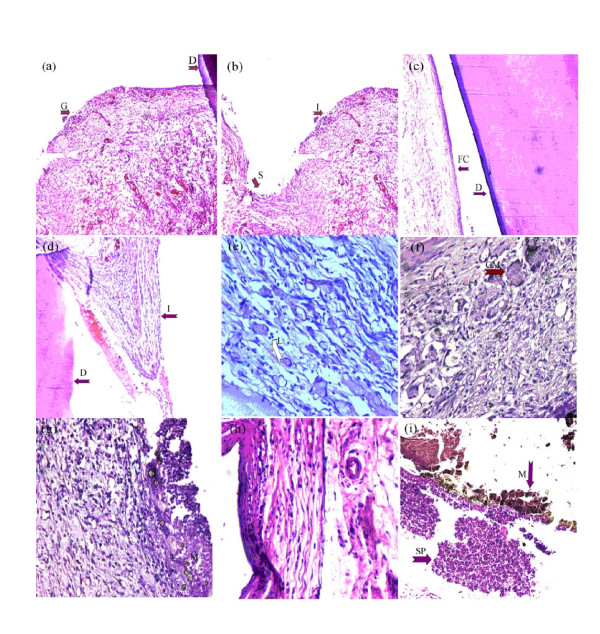
**Histologic images of inflammatory cell infiltration at the end of implanted tubes and ingrowth of granulation tissues inside the tubes in control and MTA groups (hematoxylin-eosin staining)**. (a) Highly vascular, moderately inflamed granulation tissue (G) at the dentine tube end (D) in the control group at 7 days; original magnification × 50. (b) Ingrowth of inflamed granulation tissue (I) inside the lumen of the tube in the control group at 7 days. Space previously occupied by dentine tube end is represented by (S); original magnification × 100. (c) A thin fibrous capsule (FC) surrounding the empty dentine tube (D) at 30 days; original magnification × 200 (d) Ingrowth of inflamed granulation tissue (I) inside an empty dentine tube (D) at 30 days; original magnification × 200. (e) Very mild inflammation observed in some cases of the control group at 60 days, in which lymphocytes (L) were the predominant cells; original magnification × 400. (f) Active angiogenesis with inflamed granulation tissue at the area in contact with MTA at 7 days. Multinucleated giant cell with engulfed MTA (GM); original magnification × 200. (g) Necrosis in the area adjacent to MTA material (M) at 7 days; original magnification × 200. (h) Mild inflammatory reaction in an MTA specimen at 30 days; original magnification × 400. (i) Suppuration (SP) observed in the area adjacent to MTA material (M) at the tube at 30 days; original magnification × 50.

In specimens obtained at 7 days, vascular granulation tissue was observed (Figure 1.a) that was dominated by PMNs, plasma cells and lymphocytes, with a few giant cells and macrophages. Inflamed granulation tissue that had grown into the tubes was also observed (Figure 1.b). At 30 days, mild inflammatory cell infiltrates, mainly dominated by lymphocytes and thin fibrous capsules surrounding the tubes, were observed (Figure 1.c). Inflamed granulation tissue was also noticed inside the tubes, but with less inflammatory infiltration (Figure 1.d). At day 60, a few scattered inflammatory cell infiltrates were observed (Figure 1.e), beside an increase in fibroblastic activity and a thicker fibrous capsule. No necrosis was detected.

There was a statistically significant difference in the sum of inflammatory cells between the three test periods for the control group (P value < 0.05). The inflammatory cell number was significantly higher for the 7-day than the 30-day group.

### WMTA group

**Figure 2:**
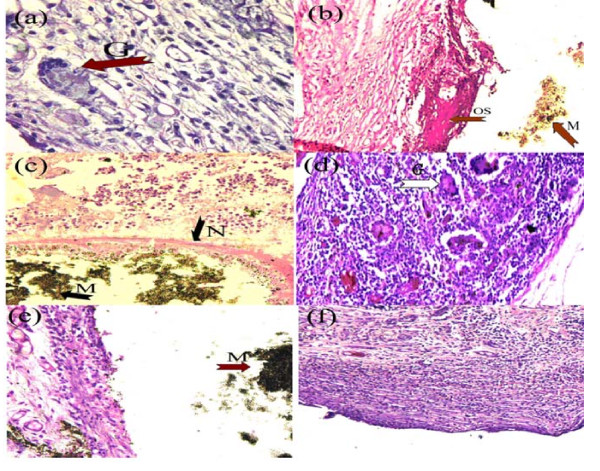
**Histologic images of inflammatory cell infiltration and tissue reaction characteristics at the end of implanted tubes in MTA and Diaket groups (hematoxylin-eosin staining)**. (a) Few macrophages and giant cells (G) observed in the MTA group at 30 days; original magnification × 400. (b) Osteoid-like material (OS) observed in the area adjacent to MTA (M) at 60 days; original magnification × 100. (c) Severe and extensive liquefactive necrosis or suppuration (N) in the area adjacent to Diaket material (M) at 7 days; original magnification × 100. (d) Giant cells (G) and macrophages in the connective tissue adjacent to Diaket at 30 days; original magnification × 200. (e) Mild inflammation observed in the area adjacent to Diaket (M) at 60 days; original magnification × 100. (f) Predominance of lymphocytes in the Diaket group at 60 days; original magnification × 50.

In specimens obtained at 7 days, necrosis surrounded by active angiogenesis and moderately inflamed granulation tissue was observed (Figure 1.h,g). The infiltrate was predominated by PMNs, lymphocytes, plasma cells, macrophages and giant cells. Similar inflammatory reactions were observed on day 30 that were characterized by necrosis and/or suppuration, surrounded by a moderate inflammatory infiltrate (Figure 1.h,i). Increased fibroblastic activity and scarce giant cells and macrophages were also detected (Figure 2.a). The inflammation was mild on day 60, and dominated by lymphocytes, macrophages and giant cells, with an increase in fibroblastic activity. Additionally, an osteoid-like material was observed adjacent to the WMTA on day 60 (Figure 2.b) in one specimen.

A statistically significant difference was found in the sum of inflammatory cells between the three test periods for this group. The highest sum of inflammatory cells was recorded for the 7-day group and the lowest for the 60-day group. However, significant differences were found only between the 7-day group and 60-day group, and between the 30-day group and the 60-day group.

### Diaket group

In sections obtained at 7 days, extensive liquefactive necrosis surrounded by highly vascularised inflamed granulation tissue was detected (Figure 2.c). The predominant inflammatory cells were PMNs, plasma cells and macrophages. At 30 days after implantation, the inflammatory reaction decreased with an increase of giant cell and macrophage infiltrates (Figure 2.d). At day 60, the inflammation was mild to moderate (Figure 2.e), and dominated by lymphocytes and plasma cells (Figure 2.f). No necrosis was observed. Increased fibroblastic activity was also noticed, but was not as pronounced as in the control and WMTA groups.

Statistically significant differences were observed between the sum of inflammatory cells among the three test periods (P < 0.05). The only statistically significant difference found was the one between the 7-day and 60-day periods (P < 0.05), with the 7-day period exhibiting the highest mean value for the sum of inflammatory cells, followed by the 30-day then the 60-day periods.

### Comparison among the groups

#### 7 days

There were statistically significant differences (KW = 7.78, p < 0.05) in inflammatory cell numbers between the three groups. The median inflammatory cell score in the Diaket group (median score of 2) was significantly higher than in the WMTA (median score of 1) and the control group (median score of 1) group (p < 0.05). Necrosis was observed in all specimens in the Diaket group and in three specimens in the WMTA group.

#### 30 days

There were statistically significant differences (KW = 6.84, p < 0.05) in inflammatory cell numbers among the three groups. The median inflammatory cell score in the Diaket group (median score of 2) was significantly higher than in the control group (median score of 1), (p < 0.05). No other significant differences were found. Necrosis was observed in only one specimen in the Diaket group and one specimen in the WMTA group.

#### 60 days

There were statistically significant differences (KW = 6.43, p < 0.05) in inflammatory cell numbers among the three groups. The inflammatory cell scores in the Diaket group (median score of 1) were significantly higher than in the WMTA (median score of 0) and the control group (median score of 0). Necrosis was not detected in any specimen obtained 60 days after implantation.

## Discussion

The histological response to endodontic materials implanted in the subcutaneous connective tissue of rats provides a preliminary source of information on their biocompatibility that is relatively uncomplicated and suitable for the routine testing of endodontic materials [29,30]. Early experiments on implantation tests involved direct insertion of the tested material into the soft tissues of experimental animals [31,32]. The disadvantage of this method was that a relatively large area of material was in contact with the tissue and, thus, the immediate reaction could be severe. Therefore, the implantation of the test material inside a carrier is recommended [12,13]. When compared with direct application of the material, this method helps to stabilize the material in place, and achieve a standardized material-tissue interface [13].

Some previous studies used silicon [30], Teflon [29,31], and polyethylene [13,33]. In this study, dentine tubes were used for subcutaneous implantation in accordance with other previous studies, to render the current investigation more clinically relevant [12,34]. One disadvantage of using human roots as carriers is the difficulty of standardization of the size of the tubes. In addition, an immunological reaction to foreign proteins may occur [31]. However, the reaction to empty dentine tubes was in agreement with previous reports which found that empty dentine tubes induced mild inflammation and formation of fibrous capsules [12,34]. The empty dentine tubes were used as a control to exclude any increase in inflammatory cell counts caused by the reaction to dentine [7,12].

The studied materials were used in the unset state to mimic the clinical situation. WMTA is difficult to handle, and this is one of its disadvantages, as it tends to smear and contaminate the working area. Therefore, meticulous attention was practiced during the placement of WMTA into the dentine tubes. In the clinical situation, WMTA has to be handled carefully to minimise this problem.

The observed reaction in the control group was in agreement with previous reports [12-14]. There was a significant continuous decrease in inflammatory response accompanied by an increase in fibroblastic activity. Although PMNs were predominant at the 7th day, they exhibited a lower mean count compared to Diaket and WMTA groups. This mean value decreased significantly at 30 and 60 days, which indicates that empty dentine tubes may have induced an initial inflammatory reaction. The inflammatory response at 7 days may be due to the toxic effects of the implanted materials, or to the trauma produced during placement of the tubes, while at 30 and 60 days, the inflammatory response may be attributed to the cumulative effect of the toxicity of the test materials.

The tissue reaction to WMTA was comparable to the findings of previous studies [12-14]. Seven-day results showed a moderate and foreign body reaction that decreased at 30 days and significantly decreased at 60 days, and was accompanied by a pronounced increase in the fibroblastic activity. This indicates an increase in the repair process and tolerability by the connective tissue. Furthermore, the osteoid-like material observed at day 60 is somewhat comparable to previous findings that described the formation of dystrophic calcification [13].

The principal compounds present in MTA are tricalcium silicate, tricalcium aluminate, tricalcium oxide and silicate oxide [2]. After reaction with tissue fluids, calcium oxide produces calcium hydroxide. The calcium hydroxide will be subsequently dissociated into Ca^+ ^and OH^-^. The diffused hydroxyl ions raise the pH and promote alkalinisation in the adjacent tissues, which favours healing [7]. Moreover, the biocompatibility of MTA was also attributed to its propensity to release calcium, and its ability to form hydroxyapatite [35].

Diaket caused an initial severe reaction, but both 30- and 60-day results showed that the inflammatory reaction decreased to a mild-to-moderate response. These observations are similar to the findings of a previous investigation [31]. Moreover, a significant decrease in the mean value of the sum of the inflammatory cells at day 60 was detected, accompanied by a minimal increase in fibroblastic activity.

This may indicate that Diaket has a tendency to induce more severe and acute initial inflammatory reaction with extensive liquefactive necrosis and/or suppuration than WMTA. At 30 days, the WMTA and Diaket groups demonstrated comparable mean inflammatory cell counts. The inflammatory reaction severity decreased significantly at day 60. Similar results were obtained in a previous animal study despite the differences in the methodologies followed compared to the current investigation [21].

The initial severe inflammatory response to Diaket could have been caused by the possible initial release of the material constituents, and when there was a decrease in the amount of these leachable substances, the inflammation decreased in severity and the material was more tolerated by tissues.

## Conclusions

In conclusion, the initial inflammatory response caused by WMTA was more favourable than the response caused by Diaket. However, with the passage of time, there was a pronounced reduction in the severity of the inflammatory response, and an increase in the fibroblastic activity in both materials. Additional experiments are necessary to confirm these observations.

## Abbreviations

WMTA: White Mineral trioxide aggregate; EDTA: ethylene diamine-tetra-acetic acid; PMNs: polymorphonuclear leukocytes

## Competing interests

The authors declare that they have no competing interests.

## Authors' contributions

AW participated in developing the methodology of the research, assisting in preparing the specimens, drafting the manuscript and statistical analysis. AM performed the experimental work, assisted in drafting the manuscript and in the examination of the specimens. HH participated in developing the methodology of the research, and has prepared and examined the specimens. All authors read and approved the manuscript final

## Pre-publication history

The pre-publication history for this paper can be accessed here:

http://www.biomedcentral.com/1472-6831/11/17/prepub
